# Crystal structure of potassium (1*S*)-d-lyxit-1-yl­sulfonate monohydrate

**DOI:** 10.1107/S2056989015014139

**Published:** 2015-07-31

**Authors:** Alan H. Haines, David L. Hughes

**Affiliations:** a School of Chemistry, University of East Anglia, Norwich NR4 7TJ, United Kingdom

**Keywords:** crystal structure, d-lyxose bis­ulfite adduct, potassium hydrogen sulfite, potassium metabisulfite, hydrogen bonding

## Abstract

The title compound, K^+^·C_5_H_11_O_8_S^−^·H_2_O [systematic name: potassium (1*S*,2*S*,3*S*,4*R*)-1,2,3,4,5-penta­hydroxy­pentane-1-sulfonate monohydrate], formed by reaction of d-lyxose with potassium hydrogen sulfite in water, crystallizes as colourless square prisms. The anion has an open-chain structure in which the S atom, the C atoms of the sugar chain and the oxygen atom of the hy­droxy­methyl group form an essentially all-*trans* chain with the corresponding torsion angles lying between 178.61 (12) and 157.75 (10)°. A three-dimensional bonding network exists in the crystal structure involving coordination of two crystallographically independent potassium ions by O atoms (one cation being hexa- and the other octa-coordinate, with each lying on a twofold rotation axis), and extensive inter­molecular O—H⋯O hydrogen bonding.

## Abstract 2

The anion has an open-chain structure in which the S atom, the C atoms of the sugar chain and the oxygen atom of the hy­droxy­methyl group form an essentially all-*trans* chain. A three-dimensional bonding network exists in the crystal structure involving coordination of two crystallographically independent potassium ions by O atoms (one cation being hexa- and the other octa-coordinate, with each lying on a twofold rotation axis), and extensive inter­molecular O—H⋯O hydrogen bonding.

## Chemical context   

In aqueous solution, the bis­ulfite anion HSO_3_^−^ exists in a complex, pH-dependent equilibrium with sulfurous acid H_2_SO_3_ and the sulfite anion SO_3_^2−^. These sulfur compounds are widely used in the preservation of foodstuffs because of their anti-oxidant and anti­microbial properties. Dissolution of sodium or potassium metabisulfite (Na_2_S_2_O_5_ or K_2_S_2_O_5_, respectively) in water affords a mixture of such compounds, along with sulfur dioxide, and they are widely used (*e.g.* as food additive E223) for their anti-oxidant, bactericidal and preservative properties. The reaction of the bis­ulfite ion with carbonyl compounds to give hy­droxy­sulfonic acids has long been known as a method of aldehyde purification; less well recognized generally is that reaction of an aldehydo-sugar, which exists predominantly in a cyclic, hemi-acetal form, with a bis­ulfite anion affords the open-chain form of the carbohydrate in which the carbonyl group has undergone addition of the sulfur nucleophile. A possible role in the stabilization of food stuffs led to early studies (Gehman & Osman, 1954 ▸) and evidence for the acyclic nature of such compounds was first provided by Ingles (1959 ▸), who reported on such adducts from d-glucose, d-galactose, d-mannose, l-arabinose and l-rhamnose. However, conclusive proof of the acyclic nature of these bis­ulfite adducts was first given through the X-ray studies of Cole *et al.* (2001 ▸) who reported the crystal structures of d-glucose- and d-mannose-derived potassium sulfonates. Later studies by X-ray crystallography on the sodium sulfon­ate derived from d-glucose (Haines & Hughes, 2012 ▸) and the potassium sulfonates from d-galactose (Haines & Hughes, 2010 ▸) and d-ribose (Haines & Hughes, 2014 ▸) proved their acyclic nature and allowed, in each case, the configuration at the newly formed chiral centre to be determined.

The crystallization of the bis­ulfite adducts of aldoses requires reactions to be conducted in concentrated solution, and success can be dependent on the particular aldose and the choice of the alkali metal ion. Thus, we have prepared the potassium adduct from l-arabinose as described by Ingles (1959 ▸), having properties in agreement with those reported, but despite prolonged efforts have not succeeded in obtaining suitable crystals for X-ray determination. Our attempts to make a crystalline potassium sulfonate from d-xylose have not been successful. In contrast, d-ribose readily afforded suitable crystals (Haines & Hughes, 2014 ▸) and we were therefore prompted to investigate the reaction of the remaining pentose, d-lyxose, with the bis­ulfite ion, from which we isolated the nicely crystalline title product (see Scheme). We report here its crystal structure.
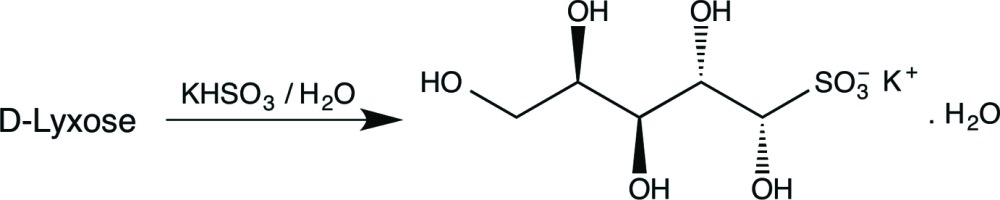


## Structural commentary   

**Figure 1:**
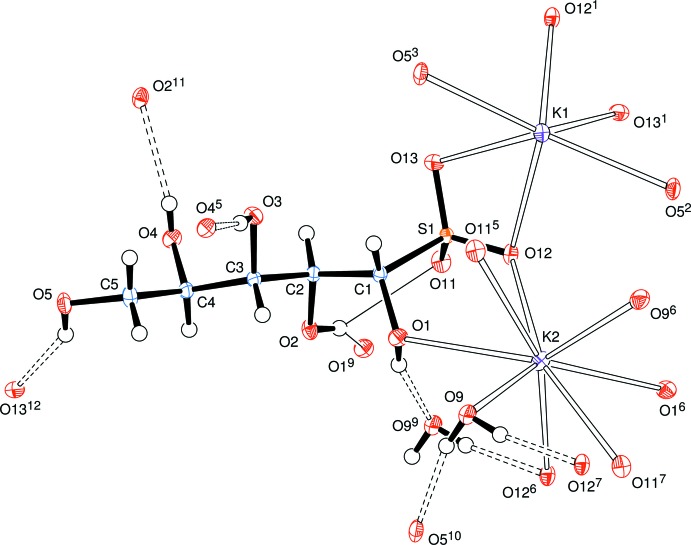
View of a mol­ecule of a d-lyxose-KHSO_3_ adduct and water mol­ecule, indicating the atom-numbering scheme. The coordination spheres of the two potassium ions (both lying on twofold rotation axes), and the hydrogen bonds (dashed lines) on the lyxose unit, are shown. Displacement ellipsoids are drawn at the 50% probability level. [Symmetry codes: (1) −*x* + 1, −*y* + 2, *z*; (2) *x* + 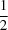, −*y* + 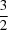, −*z* + 1; (3) −*x* + 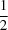, *y* + 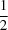, −*z* + 1; (4) −*x* + 1, −*y* + 2, *z* + 1; (5) *x*, *y*, *z* + 1; (6) −*x* + 1, −*y* + 1, *z*; (7) −*x* + 1, −*y* + 1, *z* + 1; (8) *x*, *y* − 1, *z*; (9) *x*, *y*, *z* − 1; (10) −*x* + 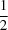, *y* − 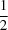, −*z* + 1; (11) −*x* + 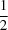, *y* + 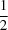, −*z*; (12) −*x* + 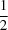, *y* − 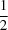, −*z*.]

The systematic name for the salt is potassium (1*S*,2*S*,3*S*,4*R*)-1,2,3,4,5-penta­hydroxy­pentane-1-sulfonate monohydrate. The anion has an open-chain structure in which the S atom, the C atoms of the sugar chain and the O atom of the hy­droxy­methyl group form an essentially all-*trans* chain with the corresponding torsion angles lying between absolute values of 178.61 (12) (for C2—C3—C4—C5) and 157.75 (10)° (for S1—C1—C2—C3). The newly formed chiral centre at C1 has the *S* configuration (Fig. 1 ▸). For each lyxose residue, all hy­droxy groups act as hydrogen-bond donors (Table 1 ▸). Atom H2*O* is involved in a bifurcated hydrogen bond to O11 in the same mol­ecule and to O1 in a neighbouring mol­ecule (at *x*, *y*, *z* − 1). Atom H1*O* is involved in hydrogen bonding to atom O9 of a water mol­ecule, the H atoms of which are hydrogen-bonded to O5 and O12 of adjoining mol­ecules. Two crystallographically independent potassium ions are present, each one lying on a twofold rotation axis, with one cation possessing a coordination sphere of six O atoms (assuming a cut-off distance of 3 Å), four coming from two different sulfonate residues and two from O atoms of hy­droxy­methyl groups. The other cation is octa­coordinate with oxygen atoms arising from two water mol­ecules, two O atoms at new chiral centres at C1, and from two pairs of O atoms from different sulfonate residues. The range of cation–oxygen bond lengths in the coordination spheres lie in the range 2.7787 (12) to 2.9855 (12) Å, but it should be noted that the designated hexa­coordinate potassium ion does have two further neighbouring O atoms at 3.1131 (12) and 3.3824 (13) Å. Variability in the coordination spheres of potassium ions in related coordination environments was observed in the d-galactose bis­ulfite (Haines & Hughes, 2010 ▸), d-glucose bis­ulfite (Cole *et al.* 2001 ▸; Haines & Hughes, 2012 ▸) and de­hydro-l-ascorbic acid bis­ulfite (Haines & Hughes, 2013 ▸) adducts, where the potassium ion is, respectively, six-, seven- and eight-coordinate.

**Figure 2:**
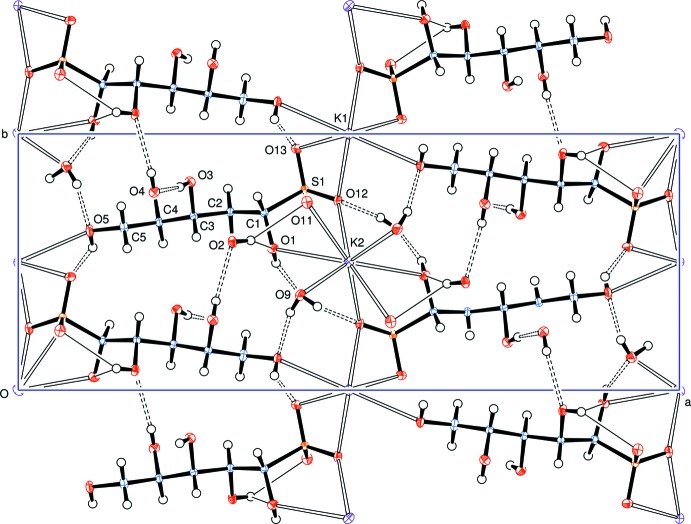
Packing diagram, viewed along the *c* axis, showing the approximately parallel alignment of the d-lyxose chains between sheets of potassium ions and water mol­ecules. Hydrogen bonds are shown as dashed lines; the fine line bonds are of bifurcated hydrogen bonds. Please note that the atoms labelled O2, O4 and O11 are eclipsing the real atoms of those names.

**Figure 3:**
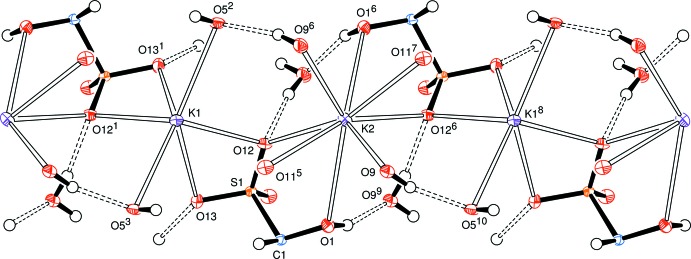
View (slightly offset from along the *c* axis) of the sheets of potassium ions which are linked through coordinating d-lyxose-sulfite anions and water mol­ecules. Symmetry codes are as in Fig. 1 ▸.

**Figure 4:**
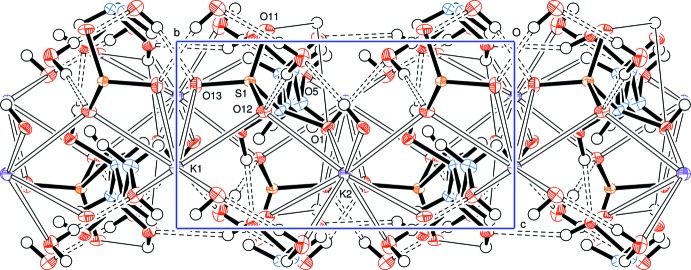
View along the *a* axis, showing the approximately parallel alignment of the d-lyxose chains.

A view along the *c* axis (Fig. 2 ▸) indicates the approximately parallel but alternating alignment of the d-lyxose chains between sheets of potassium ions and water mol­ecules, with hydrogen bonds shown as dashed bonds except for the bifurcated hydrogen bonds which are denoted by fine line bonds. Cation coordination with d-lyxose sulfite anions and water mol­ecules is depicted in Fig. 3 ▸ and a view along the *a* axis (Fig. 4 ▸) shows the approximately parallel alignment of the d-lyxose chains.

## Supra­molecular features   

A three-dimensional network exists in the crystal structure through coordination of (i) a hexa­coordinate potassium ion with O atoms from four different d-lyxose bis­ulfite residues, (ii) an octa­coordinate potassium ion with O atoms from four different d-lyxose bis­ulfite residues and two different water mol­ecules, (iii) inter­molecular hydrogen bonding between hy­droxy groups of the d-lyxose moieties, and (iv) hydrogen bonding between a water mol­ecule and two different lyxose residues. Despite spectroscopic evidence for a diastereoisomeric adduct in solution, only the 1*S* stereoisomer crystallized from the reaction mixture.

## Spectroscopic findings   

High-resolution mass spectrometry in negative-ion mode showed no significant peak for ([C_5_H_11_O_8_S_1_]^−^) at the calculated *m*/*z* of 231.0180, but a significant peak was observed at 213.0073 ([C_5_H_11_O_8_S – H_2_O] ^−^). A similar loss of water from the parent anion was observed in the case of the d-ribose adduct (Haines & Hughes, 2014 ▸). A peak at 149.0457 ([C_5_H_9_O_5_]^−^) arose from the parent sugar and the base peak was at 299.0979 ([C_10_H_19_O_10_]^−^). The latter corresponds to the ion of the product formed by reaction between the bis­ulfite adduct and d-lyxose with displacement of potassium bis­ulfite.

The ^1^H NMR spectrum of the adduct in D_2_O showed the presence the α- and β-pyran­ose forms of d-lyxose and the major and minor forms of the acyclic sulfonate in the % ratios of 35.48 : 11.29 : 48.39 : 4.84. The adduct undergoes partial hydrolysis in aqueous media; notably, it is present in a larger proportion in the more concentrated solution used for ^13^C NMR spectroscopy (see below). A large *J*_2,3_ coupling of 9.4 Hz suggests the conformation about the C2—C3 bond is similar in solution and the crystalline state.

In the ^13^C NMR spectrum, signals for C1 nuclei allow identification of the α- and β-pyran­ose forms of d-lyxose and the major (δ_C_ 82.20) and minor (δ_C_ 84.26) adducts in the ratios of 17.05 : 5.43 : 71.32 : 6.20, respectively.

## Synthesis and crystallization   

Water (0.5 ml) was added to potassium metabisulfite (0.37 g) which did not completely dissolve even on warming but which appeared to change its crystalline form as it underwent hydrolysis to yield potassium hydrogen sulfite. To this suspension was added a solution of d-lyxose (0.5 g) in water (0.35 ml), leading to immediate and complete solution of the reaction mixture. Seed crystals were obtained by evaporation of a small proportion of the solution, and these were added to the bulk of the solution which was then stored at 277 K, leading to the formation of large, well separated crystals. The mother liquor was removed with a Pasteur pipette, and the crystals were dried by pressing between filter papers to give, as a monohydrate, potassium (1*S*)-d-lyxit-1-yl­sulfonate (0.396 g, 41%), m.p. 392–400 K (with decomposition); [α]_D_ 7.1 (30 min.) (*c*, 0.75 in 9:1 H_2_O:HOAc). ^1^H NMR (D_2_O, 400 MHz, reference *Me*_3_COH at δ_H_ 1.24): δ_H_ 4.93 (*d*, *J*_1,2_ = 4.5 Hz, H-1 of α-pyran­ose), 4.86 (*d*, *J*_1,2_ = 1.5 Hz, H-1 of β-pyran­ose); signals for the major acyclic sulfonate: δ_H_ 4.70 (*d*, *J*_1,2_ = 1 Hz, H-1), 4.19 (*dd*, *J*_2,3_ = 9.4 Hz, H-2), 3.99 (*td*, *J*_3,4_ = 6.5, *J*_4,5b_ = 6.5, *J*_4,5a_ = 1.5 Hz, H-4), 3.62 (*dd*, *J*_5a,5b_ = −9.4, H-5a); for the minor acyclic sulfonate: δ_H_ 4.62 (*d*, *J*_1,2_ = 5.5 Hz, H-1); ratio of major to minor sulfonate = 10:1. ^13^C NMR (D_2_O, 100 MHz, reference *Me*_3_COH at δ_C_ 30.29): δ_C_ 94.86 (C1, β-pyran­ose), 94.70 (C1, α-pyran­ose); signals for the major acyclic sulfonate: δ_C_ 82.20 (C1), 70.45, 69.88, 69.35 (C2, C3, C4), 63.80 (C5); the minor acyclic sulfonate showed a peak at δ_C_ 84.26 (C1).

Integration of the various signals for H-1 in the ^1^H NMR spectrum, five minutes after sample dissolution, indicated the species α-pyran­ose, β-pyran­ose, major acyclic sulfonate and minor acyclic sulfonate were present in the % ratios of 35.48: 11.29: 48.39: 4.84. In the more concentrated solution prepared for ^13^C NMR the corresponding ratios were 17.05:5.43:71.32:6.20.

HRESMS (negative ion mode, measured in H_2_O/MeOH, solution) gave a peak at *m*/*z* 149.0457 ([C_5_H_9_O_5_]^−^), a significant peak at 213.0073 ([C_5_H_11_O_8_S – H_2_O] ^−^), and the base peak at 299.0979 ([C_10_H_19_O_10_]^−^). The latter corresponds to the ion of the product formed by reaction between the bis­ulfite adduct and d-lyxose with displacement of potassium bis­ulfite. No significant peak was observed for ([C_5_H_11_O_8_S_1_]^−^) at the calculated *m*/*z* of 231.0180.

## Refinement   

Crystal data, data collection and structure refinement details are summarized in Table 2 ▸. All the hydrogen atoms were located in difference maps and were refined freely.

## Supplementary Material

Crystal structure: contains datablock(s) I. DOI: 10.1107/S2056989015014139/lh5775sup1.cif

Structure factors: contains datablock(s) I. DOI: 10.1107/S2056989015014139/lh5775Isup2.hkl

Supporting information file. DOI: 10.1107/S2056989015014139/lh5775Isup3.cml

CCDC reference: 1415264

Additional supporting information:  crystallographic information; 3D view; checkCIF report

## Figures and Tables

**Table 1:** Hydrogen-bond geometry (, )

*D*H*A*	*D*H	H*A*	*D* *A*	*D*H*A*
O1H1*O*O9^i^	0.80(3)	1.90(3)	2.6716(19)	160(2)
O2H2*O*O1^i^	0.77(3)	2.35(3)	2.9810(17)	141(2)
O2H2*O*O11	0.77(3)	2.41(2)	2.9935(16)	134(2)
O3H3*O*O4^ii^	0.85(3)	1.91(3)	2.7609(17)	173(3)
O4H4*O*O2^iii^	0.76(3)	2.17(3)	2.8586(17)	151(3)
O5H5*O*O13^iv^	0.78(3)	2.03(3)	2.7152(17)	146(2)
O9H9*A*O5^v^	0.81(3)	1.95(3)	2.7426(18)	167(3)
O9H9*B*O12^vi^	0.84(3)	1.90(3)	2.7289(17)	170(3)

Symmetry codes: (i) 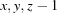; (ii) 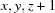; (iii) 
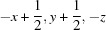
; (iv) 
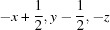
; (v) 
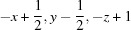
; (vi) 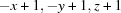.

**Table 2:** Experimental details

Crystal data	
Chemical formula	K^+^C_5_H_11_O_8_SH_2_O
*M*_r_	288.31
Crystal system, space group	Orthorhombic, *P*2_1_2_1_2
Temperature (K)	140
*a*, *b*, *c* ()	23.3536(5), 9.0434(2), 4.9939(1)
*V* (^3^)	1054.69(4)
*Z*	4
Radiation type	Mo *K*
(mm^1^)	0.74
Crystal size (mm)	0.28 0.26 0.11

Data collection	
Diffractometer	Oxford Diffraction Xcalibur 3/Sapphire3 CCD
Absorption correction	Multi-scan (*CrysAlis PRO*; Agilent, 2014 ▸)
*T*_min_, *T*_max_	0.914, 1.000
No. of measured, independent and observed [*I* > 2(*I*)] reflections	20712, 3062, 3008
*R*_int_	0.023
(sin /)_max_ (^1^)	0.703

Refinement	
*R*[*F* ^2^ > 2(*F* ^2^)], *wR*(*F* ^2^), *S*	0.018, 0.048, 1.12
No. of reflections	3062
No. of parameters	198
H-atom treatment	All H-atom parameters refined
_max_, _min_ (e ^3^)	0.30, 0.34
Absolute structure	Flack *x* determined using 1229 quotients [(*I* ^+^)(*I* )]/[(*I* ^+^)+(*I* )] (Parsons *et al.*, 2013 ▸)
Absolute structure parameter	0.023(11)

Computer programs: *CrysAlis PRO* (Agilent, 2014 ▸), *SHELXS97* (Sheldrick, 2008 ▸), *SHELXL2014* (Sheldrick, 2015 ▸) *ORTEP* (Johnson, 1976 ▸), *ORTEP-3 for Windows* and *WinGX* (Farrugia, 2012 ▸).

## supplementary crystallographic information

### Crystal data

**Table:**

K^+^·C_5_H_11_O_8_S^−^·H_2_O	*D*_x_ = 1.816 Mg m^−^^3^
*M**_r_* = 288.31	Mo *K*α radiation, λ = 0.71073 Å
Orthorhombic, *P*2_1_2_1_2	Cell parameters from 10753 reflections
*a* = 23.3536 (5) Å	θ = 3.5–32.6°
*b* = 9.0434 (2) Å	µ = 0.74 mm^−^^1^
*c* = 4.9939 (1) Å	*T* = 140 K
*V* = 1054.69 (4) Å^3^	Square prism, colourless
*Z* = 4	0.28 × 0.26 × 0.11 mm
*F*(000) = 600	

### Data collection

**Table:**

Oxford Diffraction Xcalibur 3/Sapphire3 CCD diffractometer	3062 independent reflections
Radiation source: Enhance (Mo) X-ray Source	3008 reflections with *I* > 2σ(*I*)
Graphite monochromator	*R*_int_ = 0.023
Detector resolution: 16.0050 pixels mm^-1^	θ_max_ = 30.0°, θ_min_ = 3.5°
Thin slice φ and ω scans	*h* = −32→32
Absorption correction: multi-scan (*CrysAlis PRO*; Agilent, 2014)	*k* = −12→12
*T*_min_ = 0.914, *T*_max_ = 1.000	*l* = −7→7
20712 measured reflections	

### Refinement

**Table:**

Refinement on *F*^2^	Hydrogen site location: difference Fourier map
Least-squares matrix: full	All H-atom parameters refined
*R*[*F*^2^ > 2σ(*F*^2^)] = 0.018	*w* = 1/[σ^2^(*F*_o_^2^) + (0.0253*P*)^2^ + 0.2102*P*] where *P* = (*F*_o_^2^ + 2*F*_c_^2^)/3
*wR*(*F*^2^) = 0.048	(Δ/σ)_max_ = 0.001
*S* = 1.12	Δρ_max_ = 0.30 e Å^−^^3^
3062 reflections	Δρ_min_ = −0.34 e Å^−^^3^
198 parameters	Absolute structure: Flack *x* determined using 1229 quotients [(*I*^+^)-(*I*^-^)]/[(*I*^+^)+(*I*^-^)] (Parsons *et al.*, 2013)
0 restraints	Absolute structure parameter: 0.023 (11)
Primary atom site location: structure-invariant direct methods	

### Special details

**Table:**

Experimental. CrysAlisPro, Agilent Technologies, Version 1.171.36.21 Empirical absorption correction using spherical harmonics, implemented in SCALE3 ABSPACK scaling algorithm.
Geometry. All e.s.d.'s (except the e.s.d. in the dihedral angle between two l.s. planes) are estimated using the full covariance matrix. The cell e.s.d.'s are taken into account individually in the estimation of e.s.d.'s in distances, angles and torsion angles; correlations between e.s.d.'s in cell parameters are only used when they are defined by crystal symmetry. An approximate (isotropic) treatment of cell e.s.d.'s is used for estimating e.s.d.'s involving l.s. planes.

### Fractional atomic coordinates and isotropic or equivalent isotropic displacement parameters (Å^2^)

**Table:**

	*x*	*y*	*z*	*U*_iso_*/*U*_eq_	
K1	0.5000	1.0000	0.67598 (9)	0.01296 (9)	
K2	0.5000	0.5000	0.70830 (10)	0.01438 (9)	
C1	0.37190 (6)	0.69370 (16)	0.3668 (3)	0.0102 (3)	
C2	0.32071 (6)	0.69562 (16)	0.1730 (3)	0.0099 (2)	
C3	0.26543 (6)	0.67837 (15)	0.3346 (3)	0.0104 (3)	
C4	0.21277 (6)	0.65562 (16)	0.1567 (3)	0.0112 (3)	
C5	0.15984 (6)	0.63560 (19)	0.3308 (3)	0.0150 (3)	
O1	0.38531 (5)	0.55224 (13)	0.4618 (2)	0.0142 (2)	
O2	0.32388 (5)	0.57866 (13)	−0.0172 (2)	0.0129 (2)	
O3	0.26007 (5)	0.81265 (13)	0.4827 (3)	0.0138 (2)	
O4	0.20578 (5)	0.77316 (14)	−0.0315 (2)	0.0136 (2)	
O5	0.10823 (5)	0.62153 (13)	0.1783 (3)	0.0156 (2)	
S1	0.43316 (2)	0.78331 (4)	0.21337 (7)	0.00910 (8)	
O11	0.43680 (5)	0.73320 (13)	−0.0636 (2)	0.0145 (2)	
O12	0.48337 (4)	0.74113 (13)	0.3723 (2)	0.0132 (2)	
O13	0.42133 (5)	0.94196 (12)	0.2349 (3)	0.0153 (2)	
O9	0.42697 (5)	0.37107 (14)	1.0868 (3)	0.0188 (2)	
H1	0.3619 (8)	0.756 (2)	0.520 (4)	0.008 (4)*	
H2	0.3170 (9)	0.786 (2)	0.088 (4)	0.014 (5)*	
H3	0.2694 (9)	0.598 (2)	0.456 (5)	0.015 (5)*	
H4	0.2190 (9)	0.573 (2)	0.044 (5)	0.017 (5)*	
H5A	0.1557 (9)	0.718 (3)	0.447 (5)	0.019 (5)*	
H5B	0.1647 (9)	0.553 (3)	0.444 (5)	0.019 (6)*	
H1O	0.3898 (10)	0.496 (3)	0.338 (5)	0.029 (6)*	
H2O	0.3515 (10)	0.585 (3)	−0.100 (5)	0.021 (6)*	
H3O	0.2443 (11)	0.793 (3)	0.633 (5)	0.029 (7)*	
H4O	0.2011 (12)	0.848 (3)	0.035 (6)	0.037 (8)*	
H5O	0.1126 (10)	0.557 (3)	0.075 (5)	0.023 (6)*	
H9A	0.4115 (12)	0.304 (3)	1.008 (7)	0.042 (8)*	
H9B	0.4524 (13)	0.326 (3)	1.175 (7)	0.048 (8)*	

### Atomic displacement parameters (Å^2^)

**Table:**

	*U*^11^	*U*^22^	*U*^33^	*U*^12^	*U*^13^	*U*^23^
K1	0.01062 (18)	0.01409 (18)	0.0142 (2)	−0.00037 (15)	0.000	0.000
K2	0.0152 (2)	0.01494 (19)	0.01297 (19)	0.00403 (16)	0.000	0.000
C1	0.0088 (6)	0.0117 (6)	0.0101 (6)	0.0001 (5)	0.0004 (5)	0.0014 (5)
C2	0.0087 (6)	0.0110 (6)	0.0100 (6)	0.0001 (5)	0.0000 (5)	0.0008 (5)
C3	0.0092 (6)	0.0104 (6)	0.0117 (7)	−0.0003 (5)	0.0008 (5)	0.0005 (5)
C4	0.0090 (6)	0.0116 (6)	0.0129 (7)	−0.0005 (5)	0.0004 (5)	0.0010 (6)
C5	0.0096 (6)	0.0205 (7)	0.0151 (7)	−0.0016 (5)	0.0001 (6)	0.0022 (6)
O1	0.0163 (5)	0.0135 (5)	0.0128 (5)	0.0002 (4)	−0.0024 (4)	0.0041 (4)
O2	0.0099 (5)	0.0172 (5)	0.0118 (5)	−0.0010 (4)	0.0020 (4)	−0.0032 (4)
O3	0.0140 (5)	0.0139 (5)	0.0133 (5)	−0.0014 (4)	0.0028 (4)	−0.0036 (4)
O4	0.0147 (5)	0.0146 (5)	0.0116 (5)	0.0015 (4)	−0.0002 (4)	0.0012 (4)
O5	0.0083 (5)	0.0201 (5)	0.0184 (6)	−0.0014 (4)	−0.0008 (4)	−0.0038 (5)
S1	0.00803 (14)	0.01074 (14)	0.00852 (15)	−0.00035 (11)	0.00023 (12)	0.00024 (12)
O11	0.0150 (5)	0.0200 (5)	0.0084 (5)	−0.0009 (5)	0.0011 (4)	−0.0014 (4)
O12	0.0091 (4)	0.0182 (5)	0.0124 (5)	0.0006 (4)	−0.0011 (4)	0.0013 (4)
O13	0.0160 (5)	0.0107 (4)	0.0191 (6)	−0.0006 (4)	−0.0015 (4)	0.0003 (4)
O9	0.0141 (5)	0.0159 (6)	0.0263 (6)	0.0020 (5)	−0.0060 (5)	−0.0015 (5)

### Geometric parameters (Å, º)

**Table:**

K1—O12^i^	2.8161 (12)	C2—O2	1.4233 (18)
K1—O12	2.8162 (12)	C2—C3	1.530 (2)
K1—O5^ii^	2.8505 (11)	C2—H2	0.93 (2)
K1—O5^iii^	2.8505 (11)	C3—O3	1.4274 (18)
K1—O13	2.9160 (12)	C3—C4	1.531 (2)
K1—O13^i^	2.9160 (12)	C3—H3	0.95 (2)
K1—O11^iv^	3.1131 (12)	C4—O4	1.4281 (18)
K1—O11^v^	3.1131 (12)	C4—C5	1.522 (2)
K1—O13^iv^	3.3824 (13)	C4—H4	0.95 (2)
K1—O13^v^	3.3824 (13)	C5—O5	1.4313 (18)
K1—S1^i^	3.4078 (5)	C5—H5A	0.95 (2)
K1—S1	3.4079 (5)	C5—H5B	0.94 (2)
K2—O12	2.7787 (12)	O1—H1O	0.80 (3)
K2—O12^vi^	2.7787 (12)	O2—H2O	0.77 (3)
K2—O9	2.8002 (14)	O3—H3O	0.85 (3)
K2—O9^vi^	2.8003 (14)	O4—H4O	0.76 (3)
K2—O11^v^	2.8149 (12)	O5—K1^x^	2.8505 (11)
K2—O11^vii^	2.8149 (12)	O5—H5O	0.78 (3)
K2—O1	2.9854 (12)	S1—O11	1.4579 (11)
K2—O1^vi^	2.9855 (12)	S1—O13	1.4650 (11)
K2—K1^viii^	4.5246 (1)	S1—O12	1.4665 (11)
K2—K2^v^	4.9939 (1)	S1—K1^ix^	3.6713 (5)
K2—K2^ix^	4.9939 (1)	O11—K2^ix^	2.8149 (12)
K2—H9B	3.02 (3)	O11—K1^ix^	3.1131 (12)
C1—O1	1.3998 (18)	O13—K1^ix^	3.3824 (13)
C1—C2	1.538 (2)	O9—H9A	0.81 (3)
C1—S1	1.8141 (15)	O9—H9B	0.84 (3)
C1—H1	0.98 (2)		
			
O12^i^—K1—O12	114.84 (5)	O9^vi^—K2—K1^viii^	116.12 (3)
O12^i^—K1—O5^ii^	109.61 (3)	O11^v^—K2—K1^viii^	139.74 (2)
O12—K1—O5^ii^	86.51 (3)	O11^vii^—K2—K1^viii^	42.75 (2)
O12^i^—K1—O5^iii^	86.51 (3)	O1—K2—K1^viii^	98.25 (2)
O12—K1—O5^iii^	109.61 (3)	O1^vi^—K2—K1^viii^	80.05 (2)
O5^ii^—K1—O5^iii^	150.43 (6)	K1—K2—K1^viii^	175.911 (17)
O12^i^—K1—O13	80.20 (3)	O12—K2—K2^v^	127.14 (3)
O12—K1—O13	49.96 (3)	O12^vi^—K2—K2^v^	127.14 (3)
O5^ii^—K1—O13	133.00 (4)	O9—K2—K2^v^	47.55 (3)
O5^iii^—K1—O13	72.75 (3)	O9^vi^—K2—K2^v^	47.55 (3)
O12^i^—K1—O13^i^	49.96 (3)	O11^v^—K2—K2^v^	66.13 (2)
O12—K1—O13^i^	80.20 (3)	O11^vii^—K2—K2^v^	66.13 (2)
O5^ii^—K1—O13^i^	72.76 (3)	O1—K2—K2^v^	114.35 (3)
O5^iii^—K1—O13^i^	133.00 (4)	O1^vi^—K2—K2^v^	114.35 (3)
O13—K1—O13^i^	81.87 (5)	K1—K2—K2^v^	92.044 (9)
O12^i^—K1—O11^iv^	61.01 (3)	K1^viii^—K2—K2^v^	92.044 (9)
O12—K1—O11^iv^	159.17 (3)	O12—K2—K2^ix^	52.86 (3)
O5^ii^—K1—O11^iv^	76.81 (3)	O12^vi^—K2—K2^ix^	52.86 (3)
O5^iii^—K1—O11^iv^	90.86 (3)	O9—K2—K2^ix^	132.45 (3)
O13—K1—O11^iv^	138.92 (3)	O9^vi^—K2—K2^ix^	132.45 (3)
O13^i^—K1—O11^iv^	82.96 (3)	O11^v^—K2—K2^ix^	113.87 (2)
O12^i^—K1—O11^v^	159.16 (3)	O11^vii^—K2—K2^ix^	113.87 (2)
O12—K1—O11^v^	61.01 (3)	O1—K2—K2^ix^	65.65 (3)
O5^ii^—K1—O11^v^	90.86 (3)	O1^vi^—K2—K2^ix^	65.65 (3)
O5^iii^—K1—O11^v^	76.81 (3)	K1—K2—K2^ix^	87.956 (9)
O13—K1—O11^v^	82.96 (3)	K1^viii^—K2—K2^ix^	87.956 (9)
O13^i^—K1—O11^v^	138.92 (3)	K2^v^—K2—K2^ix^	180.0
O11^iv^—K1—O11^v^	130.61 (4)	O12—K2—H9B	145.3 (6)
O12^i^—K1—O13^iv^	103.91 (3)	O12^vi^—K2—H9B	96.3 (6)
O12—K1—O13^iv^	130.41 (3)	O9—K2—H9B	16.1 (6)
O5^ii^—K1—O13^iv^	50.78 (3)	O9^vi^—K2—H9B	85.4 (6)
O5^iii^—K1—O13^iv^	102.19 (3)	O11^v^—K2—H9B	83.3 (6)
O13—K1—O13^iv^	173.46 (3)	O11^vii^—K2—H9B	59.4 (6)
O13^i^—K1—O13^iv^	104.67 (3)	O1—K2—H9B	94.0 (6)
O11^iv^—K1—O13^iv^	43.74 (3)	O1^vi^—K2—H9B	124.5 (6)
O11^v^—K1—O13^iv^	91.89 (3)	K1—K2—H9B	123.1 (6)
O12^i^—K1—O13^v^	130.41 (3)	K1^viii^—K2—H9B	60.6 (6)
O12—K1—O13^v^	103.91 (3)	K2^v^—K2—H9B	39.5 (6)
O5^ii^—K1—O13^v^	102.19 (3)	K2^ix^—K2—H9B	140.5 (6)
O5^iii^—K1—O13^v^	50.78 (3)	O1—C1—C2	113.41 (12)
O13—K1—O13^v^	104.67 (3)	O1—C1—S1	112.02 (10)
O13^i^—K1—O13^v^	173.46 (3)	C2—C1—S1	110.00 (10)
O11^iv^—K1—O13^v^	91.89 (3)	O1—C1—H1	108.2 (12)
O11^v^—K1—O13^v^	43.74 (3)	C2—C1—H1	107.6 (11)
O13^iv^—K1—O13^v^	68.79 (4)	S1—C1—H1	105.2 (11)
O12^i^—K1—S1^i^	25.01 (2)	O2—C2—C3	108.66 (11)
O12—K1—S1^i^	100.14 (3)	O2—C2—C1	111.77 (11)
O5^ii^—K1—S1^i^	89.35 (3)	C3—C2—C1	108.82 (12)
O5^iii^—K1—S1^i^	110.94 (3)	O2—C2—H2	110.9 (13)
O13—K1—S1^i^	83.10 (3)	C3—C2—H2	104.6 (13)
O13^i^—K1—S1^i^	25.29 (2)	C1—C2—H2	111.7 (13)
O11^iv^—K1—S1^i^	67.69 (2)	O3—C3—C2	105.10 (11)
O11^v^—K1—S1^i^	161.09 (2)	O3—C3—C4	110.15 (12)
O13^iv^—K1—S1^i^	102.79 (2)	C2—C3—C4	112.67 (12)
O13^v^—K1—S1^i^	153.82 (2)	O3—C3—H3	109.2 (14)
O12^i^—K1—S1	100.14 (3)	C2—C3—H3	109.4 (14)
O12—K1—S1	25.01 (2)	C4—C3—H3	110.2 (13)
O5^ii^—K1—S1	110.94 (3)	O4—C4—C5	111.81 (12)
O5^iii^—K1—S1	89.34 (3)	O4—C4—C3	111.94 (12)
O13—K1—S1	25.29 (2)	C5—C4—C3	109.69 (12)
O13^i^—K1—S1	83.11 (3)	O4—C4—H4	102.4 (14)
O11^iv^—K1—S1	161.09 (2)	C5—C4—H4	111.7 (14)
O11^v^—K1—S1	67.69 (2)	C3—C4—H4	109.1 (13)
O13^iv^—K1—S1	153.82 (2)	O5—C5—C4	113.00 (13)
O13^v^—K1—S1	102.79 (2)	O5—C5—H5A	108.0 (13)
S1^i^—K1—S1	94.639 (16)	C4—C5—H5A	109.8 (13)
O12—K2—O12^vi^	105.71 (5)	O5—C5—H5B	110.5 (13)
O12—K2—O9	130.48 (3)	C4—C5—H5B	109.7 (13)
O12^vi^—K2—O9	99.55 (3)	H5A—C5—H5B	105.5 (19)
O12—K2—O9^vi^	99.55 (3)	C1—O1—K2	119.01 (9)
O12^vi^—K2—O9^vi^	130.48 (3)	C1—O1—H1O	110.1 (19)
O9—K2—O9^vi^	95.09 (6)	K2—O1—H1O	95.8 (18)
O12—K2—O11^v^	65.36 (3)	C2—O2—H2O	110.5 (18)
O12^vi^—K2—O11^v^	154.90 (3)	C3—O3—H3O	108.4 (18)
O9—K2—O11^v^	73.70 (4)	C4—O4—H4O	113 (2)
O9^vi^—K2—O11^v^	74.59 (4)	C5—O5—K1^x^	130.19 (10)
O12—K2—O11^vii^	154.90 (3)	C5—O5—H5O	107.9 (17)
O12^vi^—K2—O11^vii^	65.36 (3)	K1^x^—O5—H5O	89.8 (17)
O9—K2—O11^vii^	74.59 (4)	O11—S1—O13	112.64 (7)
O9^vi^—K2—O11^vii^	73.70 (4)	O11—S1—O12	112.71 (7)
O11^v^—K2—O11^vii^	132.26 (5)	O13—S1—O12	111.46 (7)
O12—K2—O1	60.09 (3)	O11—S1—C1	107.93 (7)
O12^vi^—K2—O1	90.03 (3)	O13—S1—C1	104.94 (7)
O9—K2—O1	78.33 (4)	O12—S1—C1	106.60 (7)
O9^vi^—K2—O1	139.37 (4)	O11—S1—K1	142.67 (5)
O11^v^—K2—O1	65.03 (3)	O13—S1—K1	58.23 (5)
O11^vii^—K2—O1	139.10 (3)	O12—S1—K1	54.30 (5)
O12—K2—O1^vi^	90.03 (3)	C1—S1—K1	109.38 (5)
O12^vi^—K2—O1^vi^	60.10 (3)	O11—S1—K1^ix^	56.47 (5)
O9—K2—O1^vi^	139.37 (4)	O13—S1—K1^ix^	67.11 (5)
O9^vi^—K2—O1^vi^	78.33 (4)	O12—S1—K1^ix^	101.22 (5)
O11^v^—K2—O1^vi^	139.10 (3)	C1—S1—K1^ix^	151.95 (5)
O11^vii^—K2—O1^vi^	65.03 (3)	K1—S1—K1^ix^	89.650 (8)
O1—K2—O1^vi^	131.29 (5)	S1—O11—K2^ix^	130.34 (7)
O12—K2—K1	36.31 (2)	S1—O11—K1^ix^	100.55 (6)
O12^vi^—K2—K1	139.71 (3)	K2^ix^—O11—K1^ix^	99.38 (3)
O9—K2—K1	116.12 (3)	S1—O12—K2	130.00 (6)
O9^vi^—K2—K1	66.92 (3)	S1—O12—K1	100.69 (6)
O11^v^—K2—K1	42.75 (2)	K2—O12—K1	107.94 (4)
O11^vii^—K2—K1	139.74 (2)	S1—O13—K1	96.48 (6)
O1—K2—K1	80.05 (2)	S1—O13—K1^ix^	89.37 (5)
O1^vi^—K2—K1	98.25 (2)	K1—O13—K1^ix^	104.67 (3)
O12—K2—K1^viii^	139.71 (3)	K2—O9—H9A	105 (2)
O12^vi^—K2—K1^viii^	36.31 (2)	K2—O9—H9B	97 (2)
O9—K2—K1^viii^	66.92 (3)	H9A—O9—H9B	102 (3)
			
O1—C1—C2—O2	−44.09 (16)	O13—S1—O11—K2^ix^	150.63 (7)
S1—C1—C2—O2	82.24 (13)	O12—S1—O11—K2^ix^	23.46 (10)
O1—C1—C2—C3	75.92 (15)	C1—S1—O11—K2^ix^	−93.99 (9)
S1—C1—C2—C3	−157.75 (10)	K1—S1—O11—K2^ix^	83.82 (10)
O2—C2—C3—O3	−169.41 (11)	K1^ix^—S1—O11—K2^ix^	112.07 (8)
C1—C2—C3—O3	68.67 (14)	O13—S1—O11—K1^ix^	38.57 (7)
O2—C2—C3—C4	−49.45 (15)	O12—S1—O11—K1^ix^	−88.61 (6)
C1—C2—C3—C4	−171.36 (11)	C1—S1—O11—K1^ix^	153.94 (5)
O3—C3—C4—O4	60.33 (15)	K1—S1—O11—K1^ix^	−28.25 (9)
C2—C3—C4—O4	−56.67 (16)	O11—S1—O12—K2	−95.90 (9)
O3—C3—C4—C5	−64.38 (15)	O13—S1—O12—K2	136.30 (8)
C2—C3—C4—C5	178.61 (12)	C1—S1—O12—K2	22.33 (10)
O4—C4—C5—O5	52.08 (17)	K1—S1—O12—K2	124.57 (9)
C3—C4—C5—O5	176.87 (12)	K1^ix^—S1—O12—K2	−154.07 (6)
C2—C1—O1—K2	162.60 (8)	O11—S1—O12—K1	139.53 (6)
S1—C1—O1—K2	37.34 (13)	O13—S1—O12—K1	11.73 (7)
C4—C5—O5—K1^x^	159.72 (9)	C1—S1—O12—K1	−102.24 (6)
O1—C1—S1—O11	84.07 (12)	K1^ix^—S1—O12—K1	81.36 (3)
C2—C1—S1—O11	−43.04 (11)	O11—S1—O13—K1	−139.03 (6)
O1—C1—S1—O13	−155.59 (11)	O12—S1—O13—K1	−11.20 (7)
C2—C1—S1—O13	77.30 (11)	C1—S1—O13—K1	103.80 (6)
O1—C1—S1—O12	−37.26 (12)	K1^ix^—S1—O13—K1	−104.69 (4)
C2—C1—S1—O12	−164.36 (10)	O11—S1—O13—K1^ix^	−34.34 (6)
O1—C1—S1—K1	−94.52 (10)	O12—S1—O13—K1^ix^	93.50 (6)
C2—C1—S1—K1	138.37 (8)	C1—S1—O13—K1^ix^	−151.50 (5)
O1—C1—S1—K1^ix^	135.22 (9)	K1—S1—O13—K1^ix^	104.69 (4)
C2—C1—S1—K1^ix^	8.11 (18)		

Symmetry codes: (i) −*x*+1, −*y*+2, *z*; (ii) *x*+1/2, −*y*+3/2, −*z*+1; (iii) −*x*+1/2, *y*+1/2, −*z*+1; (iv) −*x*+1, −*y*+2, *z*+1; (v) *x*, *y*, *z*+1; (vi) −*x*+1, −*y*+1, *z*; (vii) −*x*+1, −*y*+1, *z*+1; (viii) *x*, *y*−1, *z*; (ix) *x*, *y*, *z*−1; (x) −*x*+1/2, *y*−1/2, −*z*+1.

### Hydrogen-bond geometry (Å, º)

**Table:**

*D*—H···*A*	*D*—H	H···*A*	*D*···*A*	*D*—H···*A*
O1—H1*O*···O9^ix^	0.80 (3)	1.90 (3)	2.6716 (19)	160 (2)
O2—H2*O*···O1^ix^	0.77 (3)	2.35 (3)	2.9810 (17)	141 (2)
O2—H2*O*···O11	0.77 (3)	2.41 (2)	2.9935 (16)	134 (2)
O3—H3*O*···O4^v^	0.85 (3)	1.91 (3)	2.7609 (17)	173 (3)
O4—H4*O*···O2^xi^	0.76 (3)	2.17 (3)	2.8586 (17)	151 (3)
O5—H5*O*···O13^xii^	0.78 (3)	2.03 (3)	2.7152 (17)	146 (2)
O9—H9*A*···O5^x^	0.81 (3)	1.95 (3)	2.7426 (18)	167 (3)
O9—H9*B*···O12^vii^	0.84 (3)	1.90 (3)	2.7289 (17)	170 (3)

Symmetry codes: (v) *x*, *y*, *z*+1; (vii) −*x*+1, −*y*+1, *z*+1; (ix) *x*, *y*, *z*−1; (x) −*x*+1/2, *y*−1/2, −*z*+1; (xi) −*x*+1/2, *y*+1/2, −*z*; (xii) −*x*+1/2, *y*−1/2, −*z*.
